# Only Missense Mutations Affecting the DNA Binding Domain of P53 Influence Outcomes in Patients with Breast Carcinoma

**DOI:** 10.1371/journal.pone.0055103

**Published:** 2013-01-24

**Authors:** Frédérique Végran, Magali Rebucci, Sandy Chevrier, Muriel Cadouot, Romain Boidot, Sarab Lizard-Nacol

**Affiliations:** Unit of Molecular Biology, Centre Georges-François Leclerc, Dijon, France; Dartmouth, United States of America

## Abstract

The presence of a *TP53* gene mutation can influence tumour response to some treatments, especially in breast cancer. In this study, we analysed p53 mRNA expression, LOH at 17p13 and *TP53* mutations from exons 2 to 11 in 206 patients with breast carcinoma and correlated the results with disease-free and overall survival. The observed mutations were classified according to their type and location in the three protein domains (transactivation domain, DNA binding domain, oligomerization domain) and correlated with disease-free and overall survival. In our population, neither p53 mRNA expression nor LOH correlated with outcome. Concerning *TP53* mutations, 27% of tumours were mutated (53/197) and the presence of a mutation in the *TP53* gene was associated with worse overall survival (*p = *0.0026) but not with disease-free survival (*p* = 0.0697), with median survival of 80 months and 78 months, respectively. When alterations were segregated into mutation categories and locations, and related to survival, tumours harbouring mutations other than missense mutations in the DNA binding domain of P53 had the same survival profiles as wild-type tumours. Concerning missense mutations in the DNA binding domain, median disease-free and overall survival was 23 months and 35 months, respectively (*p = *0.0021 and *p*<0.0001, respectively), compared with 78 and 80 months in mutated tumours overall. This work shows that disease-free and overall survival in patients with a frameshift mutation of *TP53* or missense mutation in the oligomerization domain are the same as those in wild-type *TP53* patients.

## Introduction

The *TP53* gene is located on chromosome 17p13. The gene is composed of 19180 bp, spanning 11 exons and 10 introns. The coding sequence starts in the 2^nd^ exon and ends in the 11^th^, giving rise to a 393-amino-acid protein. This 53 kDa protein can be schematically divided into three main domains: the transactivation domain, the DNA binding domain and the oligomerization domain. Each domain plays an important role in P53 functions. Arising from the DNA binding domain, the two other domains undergo a number of post-translational modifications through phosphorylation, acetylation, methylation, ubiquitinylation and sumoylation. The transactivation domain, encoded by exons 2 and 3 [1] is serine and threonine-rich and the site of phosphorylation by ATM, ATR or Chk2 [2], which induces protein activation. This 1^st^ domain permits the interaction of P53 with numerous proteins such as CBP, CREB, MDM2 and p300 [3]. The DNA binding domain is encoded by exons 5 to 8 [1] and recognizes a consensus sequence in some promoter sequences. The majority of mutations occur in this domain [4]. Finally, the oligomerization domain is encoded by exons 9 and 10 [1]. Thanks to this domain, P53 is able to interact with itself to form an active tetramer, but it can also interact with other proteins such as BRCA1 and Rad51.

The *TP53* gene is described as the most mutated gene with a frequency of about 50% in cancer, with a specific frequency of 25% in breast cancer [5]. These mutations may induce partial or complete function loss or function gain of the monomer. As a consequence, the stability of the *TP53* gene sequence is very important for the function of P53 protein. The prognostic significance of *TP53* mutations has often been studied [6]–[7], but the impact of mutated P53 protein domains on outcomes remains controversial in breast cancer.

In this work, we analysed p53 mRNA expression, LOH in 17p13 and *TP53* mutation in a population of 206 patients. These data and the influence of the different types of *TP53* mutations and locations were then correlated with disease-free and overall survival in patients followed for 15 years.

## Materials and Methods

### Patients, Samples and DNA Extraction

We studied a population of 206 patients with primary breast carcinoma (Table 1). The samples used for this study were obtained before any form of treatment, during the period going from 1991 to 2007 at the Centre Georges François Leclerc, Dijon, France. The clinical history of the patients included in the study was well-known. The study was conducted in accordance with the Declaration of Helsinki and approved by the ethics committee of Centre Georges-François Leclerc, the Comité Consultatif de Protection des Personnes en Recherche Biomédicale de Bourgogne. Written informed consent was obtained from all patients prior to enrolment. Only samples with ≥50% of tumour cells were included in further analyses. DNA was extracted from tissue samples using the phenol-chloroform method or with TRIzol^®^ reagent (Invitrogen). The quantity and purity of DNA were assessed spectrophotometrically at 260 and 280 nm ([the A_260_/A_280_ ratio of pure DNA is higher than 1.7).

**Table 1:** Clinical details of studied population.

Clinical parameters	Total		Not treated		Anthracycline based regimen		Trastuzumab based regimen	
	N	[% mutated]	n	% mutated	n	% mutated	n	% mutated
**Age**								
≤50	97	[28]	8	[13]	69	[23]	20	[50]
>50	109	[17]	32	[6]	59	[22]	18	[17]
NA	0	[0]	0	[0]	0	[0]	0	[0]
**Hormonal receptors**								
ER −	81	[36]	4	[25]	61	[33]	16	[50]
ER +	124	[13]	36	[6]	66	[14]	22	[23]
PR −	98	[31]	12	[17]	67	[28]	19	[47]
PR +	107	[14]	28	[4]	60	[17]	19	[21]
NA	1	[0]	0	[0]	1	[0]	0	[0]
**Grade**								
2	21	[5]	11	[9]	9	[0]	1	[0]
3	100	[17]	21	[10]	57	[18]	22	[23]
4	74	[34]	6	[0]	54	[33]	14	[50]
NA	11	[0]	2	[0]	8	[13]	1	[100]
**Nodal status**								
Negative	91	[22]	40	[8]	39	[33]	12	[33]
Positive	113	[22]	0	[0]	87	[18]	26	[35]
NA	2	[0]	0	[0]	2	[0]	0	[0]

NA: Not Available.

### RNA Extraction, cDNA Synthesis

Total RNA was extracted from tissue samples by using the acid phenol-guanidium method or with TRIzol® reagent as described previously [8]. The quantity and purity of RNA were assessed spectrophotometrically at 260 and 280 nm (the A_260_/A_280_ ratio of pure RNA is higher than 1.8). The quality of RNA extracts was determined by electrophoresis through agarose gel, staining with ethidium bromide, and visualization of the 18S and 28S bands under UV light. One microgram of total RNA was reverse transcribed in 20 µl of reverse transcriptase reaction as described previously [9].

### Real-time Quantitative PCR

The real-time quantitative PCR was performed on ABI PRISM 7300 (Applied Biosystems) by using the Taqman® method as described previously [8]. Expression of Ki-67, p53 and c-myc was quantified by using the Taqman® Gene Expression Assays Hs01032443_m1, Hs00153340_m1 and Hs00153408_m1 (Applied Biosystems), respectively. Survivin expression was studied as described previously [10].

### Analysis of the TP53 Sequence and Determination of Loss Of Heterozygosity [LOH] in 17p13

The *TP53* gene was analysed in 197 tumours as described previously [10]. Sequence analysis was first performed on DNA in parallel with LOH determination. In tumours with both *TP53* mutation and LOH, cDNA sequencing was carried out to know whether the LOH influenced the expressed allele. In these cases, the tumour was classified as mutated only if p53 cDNA was mutated. All detected mutations were present in the IARC database and no new *TP53* gene SNP was observed.

### Statistical Analysis

Statistical analysis was performed with Statview 5.0 software. Correlations were analysed by using the Mann-Whitney or Kruskall-Wallis test. Only tests with *p*<0.05 were considered significant.

Overall survival was defined as the interval between the diagnosis and the last follow-up or death. Disease-free survival was defined as the time between the date of diagnosis and the date of distant metastases or local recurrence or death, whichever came first, or the last follow-up. Survival curves were generated using the Kaplan-Meier method, and the significance of differences between dichotomized patient groups was obtained by the Mantel-Cox log rank test. Only tests with *p*<0.05 were considered significant.

## Results

### p53 Expression and LOH in 17p13 Correlated with Cell Proliferation

**Figure 1:**
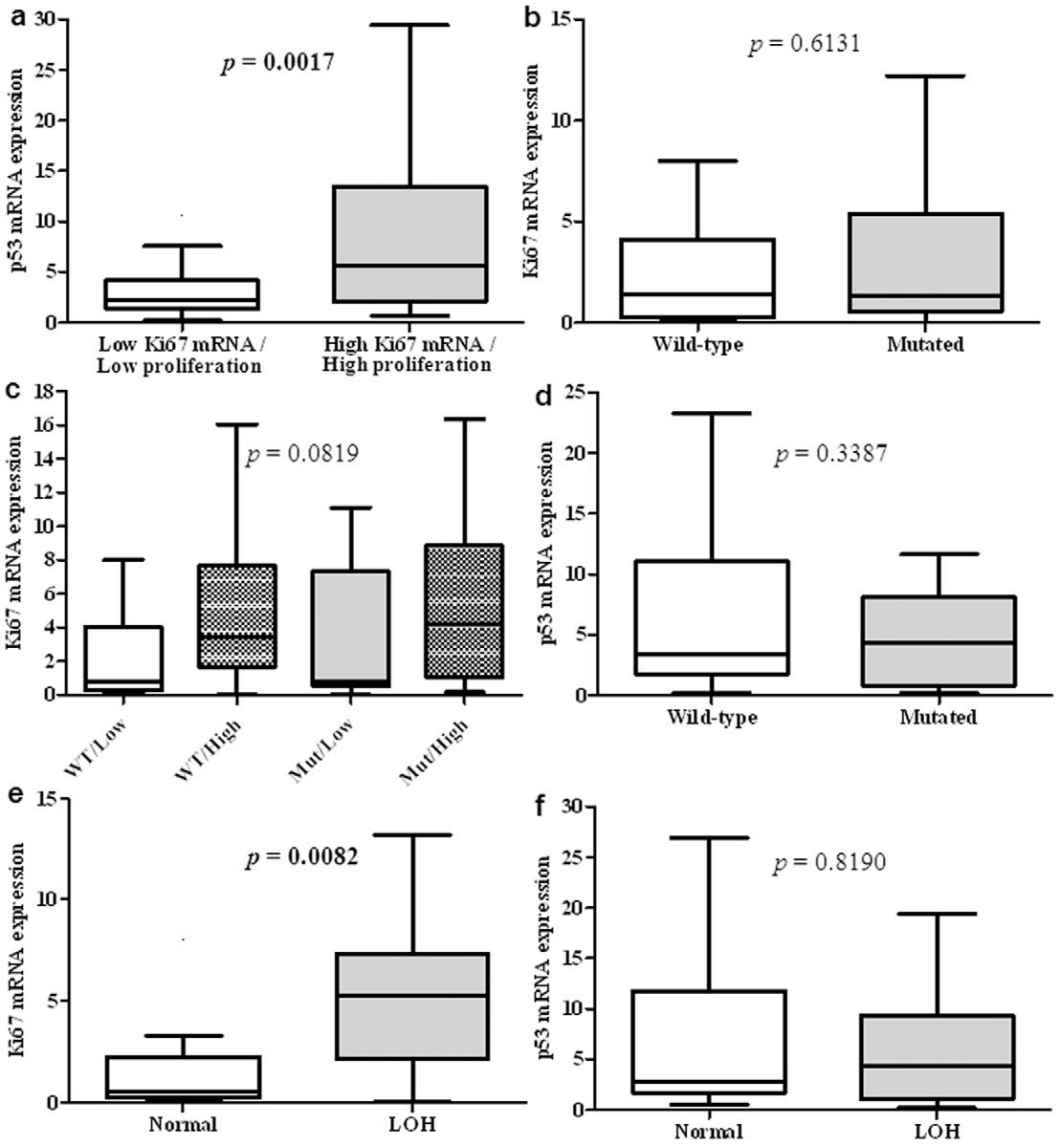
Correlations with proliferation status of tumours. a. Box plot corresponding to p53 mRNA expression depending on proliferation status. **b.** Box plot corresponding to proliferation rate depending on *TP53* mutation status. **c.** Box plot corresponding to proliferation rate depending on TP53 mutation status coupled with level of p53 mRNA expression. **d.** Box plot corresponding to proliferation rate depending on *LOH* in 17p13. **e.** Box plot corresponding to p53 mRNA expression depending on *TP53* mutation status. **f.** Box plot corresponding to p53 mRNA expression depending on *LOH* in 17p13. *p*<0.05 was considered significant.

In our population of 206 patients, we analysed the transcriptional expression of p53 by real-time quantitative PCR and the status of the *TP53* gene by sequencing and LOH analyses. In parallel, Ki-67 mRNA expression was analysed as an indicator of tumour proliferation [11]. First, we found that p53 expression significantly increased (*p* = 0.0017) with cell proliferation (Figure 1a). In parallel, we also correlated the expression of c-myc and survivin with proliferation, and we found that p53, c-myc and survivin expression was significantly increased in highly proliferative cells (*p* = 0.0001 and *p* = 0.0102, respectively, data not shown), whereas the presence of a p53 mutation was not associated (*p* = 0.6131) with higher proliferation (Figure 1b). We then classified tumours according to their *TP53* mutational status and p53 expression level. This classification revealed no correlations (*p* = 0.0819) between mutations with high expression and proliferation (Figure 1c). Moreover, the presence of a *TP53* mutation was not associated (*p* = 0.3387) with an increase or a decrease in p53 mRNA expression (Figure 1d). However, the presence of LOH correlated significantly with proliferation (*p* = 0.0082) (Figure 1e) but not with a decrease in p53 mRNA expression (*p* = 0.8190) (Figure 1f).

### Only Missense Mutations in the DNA Binding Domain of P53 are Deleterious for Outcomes

**Figure 2:**
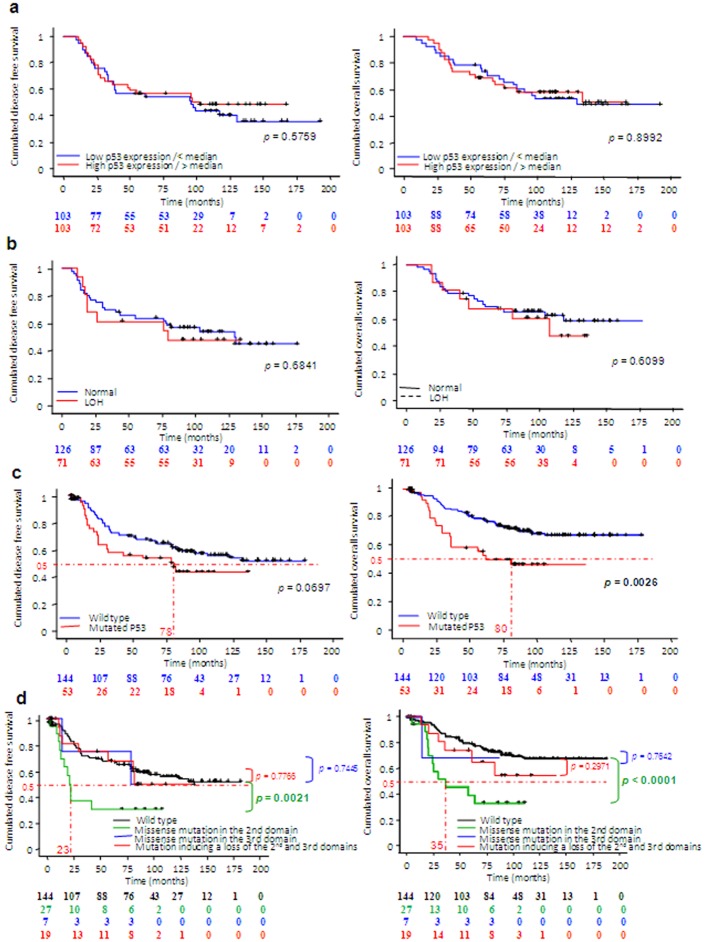
Influence of *TP53* mutations on outcomes. a. Kaplan-Meier curves of disease-free (left) and overall (right) survival in patients categorized on different categories of p53 mRNA expression. **b.** Kaplan-Meier curves of disease-free (left) and overall (right) survival in patients categorized on different categories of LOH in 17p13. **c.** Kaplan-Meier curves of disease-free (left) and overall (right) survival in patients categorized on *TP53* gene mutation status. Dotted red lines correspond to median disease-free or overall survival. **d.** Kaplan-Meier curves of disease-free (left) and overall (right) survival in patients categorized on different kinds and location of *TP53* mutation. Dotted red lines correspond to median disease-free or overall survival. *p*-values were calculated in relation to wild-type population (green: *vs.* missense mutation in the 2^nd^ domain, blue: *vs.* missense mutation in the 3^rd^ domain; red: *vs.* mutation inducing a loss of the 2^nd^ and 3^rd^ domains). Censored patients are represented on the curves by black crosses. Number at risk are presented below graphs. Only *p*<0.05 was considered significant.

We decided to study the impact of p53 mRNA expression, *TP53* LOH and mutational status on outcomes. It appeared that neither the transcriptional expression of p53 (Figure 2a), nor the presence of LOH (Figure 2b) had an impact on disease-free or overall survival. However, the presence of a mutation in the *TP53* gene significantly decreased (*p* = 0.0026) overall survival (Figure 2c, right panel) but not disease-free survival (*p = *0.0697) (Figure 2c, left panel).

**Figure 3:**
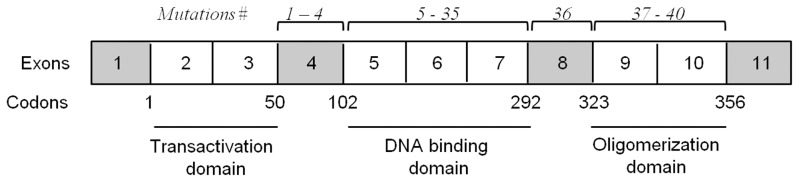
Schematic representation of P53 protein. Its 3 domains and corresponding coding exons are represented. Mutations of Table 2 appear at the top of coding exons.

Among the 197 tumours analysed, 144 had wild-type *TP53* and 53 (27%) harboured an expressed *TP53* mutation. Among these 53 mutations, one (2%) missense mutation was located outside the P53 domains (IVS5+2), 27 (51%) missense mutations were present in the DNA binding domain, six (11%) missense mutations were in the oligomerisation domain and 19 (36%) mutations were frameshift mutations in the DNA binding domain that induced a loss of both the DNA binding and oligomerisation domains (see Table 2 for details, and Figure 3). As p53 domains are known to play specific roles, we carried out Kaplan-Meier tests to estimate the impact on outcome of wild-type *TP53*, missense mutations in the 2^nd^ domain, missense mutations in the 3^rd^ domain, and mutations that induced the loss of the 2^nd^ and 3^rd^ domains. It appeared that for disease-free survival (Figure 2d, left panel) and overall survival (Figure 2d, right panel), missense mutations occurring in the DNA binding domain were significantly associated with worse survival (*p* = 0.0021, and *p*<0.0001, respectively). In contrast, neither missense mutations in the oligomerization domain, nor loss of both the 2^nd^ and 3^rd^ domains significantly affected survival as compared with wild-type *TP53* (Figure 2d). This observation suggests that these kinds of mutations could not be considered deleterious for either disease-free or overall survival.

**Table 2:** Detail of observed *TP53* gene mutations.

#	DNA Mutation	Protein effect	Impact
1	c.161_162insT	p.T55HfsX2	Loss of the 2^nd^ and 3^rd^ domain
2	c.265delT	p.S90PfrX32	Loss of the 2^nd^ and 3^rd^ domain
3	c.273G>A	p.W91X	Loss of the 2^nd^ and 3^rd^ domain
4	c.282_283insGCCCCTG	p.S95AfrX55	Loss of the 2^nd^ and 3^rd^ domain
5	c.321C>G	p.Y107X	Loss of the 2^nd^ and 3^rd^ domain
6	c.322_327del	p.G108_F109del	Mutation of DNA binding domain
7	c.327_328insACGGTTTCCGT	p.R110TfsX16	Loss of the 2^nd^ and 3^rd^ domain
8	c.358A>G	p.K120Q	Mutation of DNA binding domain
9	c.377A>G	p.Y126C	Mutation of DNA binding domain
10	c.381delC [n = 2]	p.P128LfrX41	Loss of the 2^nd^ and 3^rd^ domain
11	c.401T>G	p.F134C	Mutation of DNA binding domain
12	c.423C>G	p.C141W	Mutation of DNA binding domain
13	c.444delT	p.S149PfsX20	Loss of the 2^nd^ and 3^rd^ domain
14	c.470T>A	p.V157N	Mutation of DNA binding domain
15	c.524G>A [n = 3]	p.R175H	Mutation of DNA binding domain
16	c.530C>G	p.P177R	Mutation of DNA binding domain
17	c.569C>T [n = 2]	p.P190L	Mutation of DNA binding domain
18	c.581T>G	p.L194R	Mutation of DNA binding domain
19	c.584T>C	p.I195T	Mutation of DNA binding domain
20	c.586C>T [n = 3]	p.R196X	Loss of the 2^nd^ and 3^rd^ domain
21	c.610G>T	p.E204X	Loss of the 2^nd^ and 3^rd^ domain
22	c.637C>T	p.R213X	Loss of the 2^nd^ and 3^rd^ domain
23	c.646_647insTTA	p.S215_V216insL	Mutation of DNA binding domain
24	c.695T>C	p.I232T	Mutation of DNA binding domain
25	c.701A>G	p.Y234C	Mutation of DNA binding domain
26	c.715_737del	p.N239RfsX96	Loss of the 2^nd^ and 3^rd^ domain
27	c.733_735del	p.G245del	Mutation of DNA binding domain
28	c.734G>A	p.G245N	Mutation of DNA binding domain
29	c.742C>T	p.R248W	Mutation of DNA binding domain
30	c.746_755del	p.R249TfsX92	Loss of the 2^nd^ and 3^rd^ domain
31	c.817C>T	p.R273C	Mutation of DNA binding domain
32	c.818G>A [n = 5]	p.R273H	Mutation of DNA binding domain
33	c.821T>C	p.V274A	Mutation of DNA binding domain
34	c.868delC	p.R290AfsX54	Loss of the 2^nd^ and 3^rd^ domain
35	c.874_880del	p.K292SfsX50	Loss of the 2^nd^ and 3^rd^ domain
36	c.950delA	p.Q317RfsX27	Mutation of Oligomerization domain
37	c.967_1040del	p.D324GfsX33	Mutation of Oligomerization domain
38	c.1009C>T	p.R337C	Mutation of Oligomerization domain
39	c.1024C>T [n = 3]	p.R342X	Mutation of Oligomerization domain
40	IVS5+2	/	No impact

Finally, when we focused on median survival, we found that median disease-free survival and median overall survival in patients with mutated tumours was 78 months (Figure 2c, left panel) and 80 months (Figure 2c, right panel), respectively. Concerning the group with missense mutations in the DNA binding domain, median disease-free survival was about 23 months (Figure 2d, left panel), less than one third of that in mutated tumours overall. Median overall survival in this group was 35 months (Figure 2d, right panel), less than half that in mutated tumours overall. These results were particularly surprising as our population had node-negative tumours and locally-advanced breast tumours, which were managed using different treatments involving either anthracyclines in combination with 5-fluorouracil+cyclophosphamide, or docetaxel, or trastuzumab in combination with docetaxel (+/− carboplatine).

## Discussion

The *TP53* gene is the most altered gene in cancer with more than 2500 listed mutation points [12] and more than 24000 published mutations. In this work, we first studied the correlation between p53 expression, LOH, and/or mutation and the proliferation status of tumour cells [Ki67 mRNA expression]. It appeared that both p53 mRNA expression and LOH correlated positively with cell proliferation, whereas p53 mutations did not. Moreover, LOH did not negatively affect the mRNA expression of p53. Our results confirmed a study of Merlo *et al.* [13] in which the presence of LOH in 17p13 was associated with a high proliferation index for cancer cells, suggesting that a gene located in 17p13 could regulate cell proliferation. The same observations were obtained in astrocytic tumours in which LOH at 17p13 was associated with a higher cell proliferation [14]. Our study showed that the gene concerned was not *TP53* as its expression was not affected by LOH in 17p13. Among the 47 genes located in the 17p13 locus, besides *TP53* gene, 3 genes could be involved in the high cell proliferation rate induced by LOH: *Claudin-7*, *SERPINF1*, and *SMYD4*. The reduced expression of *Claudin-7* gene was correlated with a strong invasion, migration and metastasis ability of cancer cells [15]–[16]. Concerning *SERPINF1* gene, a suspected tumour suppressor gene, its forced expression induced a slower rate of cell proliferation [17], suggesting that a decrease of Serpinf1 may be associated with an increased cell proliferation. Finally, the *SMYD4* gene was recently identified as a potential tumour suppressor gene in breast cancer [18]. The disruption of one allele [LOH] induced tumourigenesis and high proliferation of cells.

p53 mRNA expression was higher in highly proliferative cells than in slightly proliferative cells. The same association was also found for c-myc and survivin expression. These results could indicate that highly proliferative cells exhibit general gene expression deregulation rather than specific gene expression deregulation. In our study, only *TP53* mutations correlated with outcomes, and especially DNA binding domain missense mutations. Of all known *TP53* mutations, 90% are located in the DNA binding domain of the protein, where, for the most part, they alter the capacity of the protein to bind DNA. In our study, we detected a *TP53* gene mutation in 27% [53/197] of cases, which is in accordance with the specific frequency of *TP53* gene mutations in breast cancer [5]. Moreover, 87% of the mutations detected were located in the DNA binding domain [46/53], and the others [13%] were in the oligomerization domain. It is accepted that both disease-free and overall survival in patients with a *TP53* gene mutation were shorter than in patients with wild-type p53 [6]; [19]–[28]. Nevertheless, a study reported that patients with a mutation in the DNA binding domain showed similar survival to patients with wild-type p53 [29]. Contrary to Powell *et al.*, we highlighted, by analysing the type and the location of mutations, that only missense mutations in the DNA binding domain of p53 were deleterious for survival. Our results seem to be confirmed by a recent study which showed that missense mutations affected survival of breast cancer cell lines [30]. In contrast, a very recent work undermined the dogma about the good prognosis of wild-type p53 by demonstrating that mutant P53 tumours had a better apoptotic response to anthracycline-based chemotherapy [31]. Despite the large proportion [65%] of patients treated with anthracyclines in our population, we found that a missense mutation in the DNA binding domain was not beneficial for outcomes. Indeed, we showed that missense mutations in the oligomerization domain or frameshift mutations in the DNA binding domain had no impact on either disease-free or overall survival.

The frameshift mutations in the 2^nd^ domain induced the loss of a part of the DNA binding domain and the entire oligomerization domain due to the appearance of a Stop codon. This kind of mutation accounted for 41% of DNA binding domain mutations. Based on these observations, it seems that about 50% (26/53) of *TP53* mutations (Table 2) could have no impact on survival in breast cancer. This absence of any impact could be explained by the heterozygosity of detected mutations (despite the simultaneous presence of a *TP53* mutation and LOH in a few cases, all of the expressed mutations were heterozygous). The loss of the oligomerization domain due to a missense mutation or a frameshift mutation in the DNA binding domain induces a truncated P53 protein that is unable to form tetramers. Moreover, the absence of the oligomerization domain suppresses the activity of the P53 monomer [32]. Thus, only wild-type P53 proteins are present in tetramers and only normal P53 monomers are active. This explains why these mutations had no impact on survival. Missense mutations in the DNA binding domain were deleterious, even when heterozygous, because a mutated P53 monomer is able to neutralize 75% of P53 tetramer [33]. This phenomenon is probably due to the co-translational formation of P53 dimers [34].

The *TP53* gene gives rise to numerous alternative splice variants [35]–[36]. Recently, Bourdon *et al.* [37] showed that patients with a *TP53* mutation associated with mRNA expression of the p53γ isoform had the same good prognosis as patients with wild-type p53. Nevertheless, this is paradoxical as the p53γ isoform possesses the transactivation and DNA binding domains of P53 but lacks the oligomerization domain [36]. In our work, the missense mutations in the DNA binding domain, which we linked to a poor prognosis, affect all P53 isoforms. This may explain why we found that only these *TP53* gene alterations had an impact on outcomes.

Finally, it would be interesting to investigate whether our findings could be applied to other cancers such as head and neck squamous cell carcinoma, for example, in which *TP53* mutations have an impact on survival [38], and leukemia in which missense mutations in the DNA binding domain of P53 are associated with poor survival [39]. Our classification of *TP53* gene mutations, obtained by direct sequencing, could be used in clinical practice to orientate the follow-up of patients during the remission phase. Patients with missense mutations in the DNA binding domain of P53 should be more closely monitored than patients with wild-type P53 tumours or those with a P53 mutation that does not alter wild-type P53 functions.
